# Epidemiology of uveitis in a US population-based study

**DOI:** 10.1186/s12348-018-0148-5

**Published:** 2018-04-17

**Authors:** Marta Mora González, Marissé Masís Solano, Travis C. Porco, Catherine E. Oldenburg, Nisha R. Acharya, Shan C. Lin, Matilda F. Chan

**Affiliations:** 1 Department of Ophthalmology, University of California, San Francisco, 10 Koret Way, San Francisco, CA 94143 USA; 2 F.I. Proctor Foundation, University of California, San Francisco, 513 Parnassus Avenue, San Francisco, CA 94122 USA; 3 Department of Epidemiology & Biostatistics, University of California, San Francisco, 550 16th Street, San Francisco, CA 94158 USA

**Keywords:** Epidemiology, Uveitis, Prevalence, Cigarette smoking, Arthritis, Ankylosing spondylitis, Ulcerative colitis, Crohn’s disease

## Abstract

**Background:**

The purpose of this study is to assess the relationship between self-reported uveitis and purported demographic and clinical risk factors, using an American adult population extracted from the National Health and Nutrition Examination Survey (NHANES) for the years 2009 and 2010. This is a cross-sectional, population-based study using a sample of 5106 subjects between 20 and 69 years old. The main outcome for our study was the self-report of a diagnosis of uveitis. The demographic analysis included age, gender, and ethnicity. Potential predictors were having a diagnosis of ankylosing spondylitis (AS), ulcerative colitis (UC), or Crohn’s disease (CD); a history of cigarette smoking; vitamin D deficiency; and different mental health measures. Univariate and multivariate analyses were conducted using RStudio.

**Results:**

Of the 5106 participants, 27 had reported a diagnosis of uveitis, showing an adjusted prevalence of 5.4 per 1000 subjects (95% CI 3.4–8.5/1000). Increased age was associated with higher uveitis prevalence in the multivariate analysis (odds ratio [OR] = 1.04, 95% CI 1.01–1.07; *p* = 0.02). Positive smoking history was reported in 59.2% of patients. Multivariate analysis comparing smoking with the presence of uveitis showed an OR of 3.18 (95% CI 1.59–6.37; *p* = 0.003), adjusting for age and gender. Moreover, 11.1% of the participants from the uveitis group self-reported a diagnosis of AS and 11.7% informed a diagnosis of UC and 7.1% of CD. The ORs were of 16.64 (95% CI 3.64-76.09; *p* = 0.001), 11.34 (95% CI 2.69-47.88; *p* = 0.003), and 22.16 (95% CI 2.64-186.17; *p* = 0.007), respectively when compared with the non-uveitis group in the multivariate analysis.

**Conclusions:**

Age, cigarette smoking, AS, UC, and CD are positively associated with self-reported uveitis. There is previous evidence that smoking and female gender are positive risk factors for uveitis, as well as evidence that HLA-B27-positive spondyloarthritides have the highest association with non-infectious uveitis in the adult population in North America and Europe. However, there are no prior studies that have utilized a representative US population-based sample to validate these findings. The present study supports smoking as a risk factor, which has clinical relevance since this is a modifiable habit.

## Background

Uveitis is a sight-threatening inflammatory disease affecting the uveal layer of the eye. It is responsible for an estimated 30,000 new cases of legal blindness annually in the USA [1, 2] and accounts for about 10–15% of all cases of total blindness in the country [1, 3, 4].

Approximately 30% of cases of uveitis are idiopathic and have an unknown etiology [3, 5–7]. On the other hand, an underlying systemic illness is estimated to cause 25 to 50% of the cases, resulting in a non-infectious uveitis, which is more common in developed countries compared with the infectious type [3, 8–12].

Among the non-infectious forms of uveitis, patients’ demographics strongly influence the distribution and prevalence of disease. The HLA-B27-positive spondyloarthritides, such as ankylosing spondylitis (AS), reactive arthritis (ReA), enteropathic arthropathy (EnA), and psoriatic arthritis (PsA), are the most common systemic immune disorders associated with anterior uveitis in the adult population in North America and Europe [5, 13–15]. Other disorders known to be associated with uveitis are Behçet’s disease, sarcoidosis, systemic lupus erythematosus, and multiple sclerosis [5, 16].

Risk factors for the development of uveitis have been previously reported. It is well established that there is a higher incidence in women compared with men [17] and that the risk increases with older age [17, 18], although the later has not been uniformly supported by studies [1, 10, 14, 18–23]. Smoking has also been recently reported to be positively associated with the development of uveitis [24, 25], as well as being a risk factor for active disease [26]. A positive correlation has also been observed between vitamin D deficiency and non-infectious uveitis [27, 28].

The National Health and Nutrition Examination Survey (NHANES) is a program of studies designed to assess the health and nutritional status of adults and children in the USA that has been validated and widely used for different disease analyses [29, 30]. In ophthalmology, NHANES has provided epidemiological data on macular degeneration, glaucoma, and refractive errors [31–34].

Previous studies have assessed the epidemiology of uveitis in the USA. One of them was based on insurance companies’ information and focused on non-infectious uveitis, and the other one included only a population sample from a specific area in California [12, 18]. The significant advantages of a NHANES-based study are the use of the survey data to produce representative results of the entire US population and the ability to examine potential demographic and clinical risk factors for uveitis at a nationwide level.

The purpose of our present study is to determine the association between self-reported uveitis in an adult American population and potential demographic and clinical risk factors, as well as the prevalence of the disease in the USA, using a nationwide database.

## Results

From the 5106 interviewed US residents, 27 self-reported a diagnosis of uveitis, indicating a prevalence of the disease of 5.4/1000 (95% CI 3.4–8.5/1000) in our adult American population within an age range of 20 to 69 years old. Out of the subjects with self-reported uveitis (*n* = 27), 19 (70%) reported having received treatment. The demographic characteristics of the uveitis group compared with the non-uveitis group can be found in Table 1. The results of the multivariate analysis are shown in Table 2.

**Table 1:** Demographic and clinical characteristics of the population

Characteristic	No self-reported uveitis (*n* = 5106)	Self-reported uveitis (*n* = 27)	*p* value^a^
Age mean (years) ± standard deviation	44 ± 14	53 ± 13	**<** 0.001*
Female sex (*n* (%))	2630 (51.5)	17 (63.0)	0.2
Ethnicity (*n* (%))			
Mexican	1031 (20.2)	2 (7.4)	0.1
Other Hispanic	572 (11.2)	6 (22.2)	
Non-Hispanic white	2247 (44.0)	10 (37.1)	
Non-Hispanic black	960 (18.8)	6 (22.2)	
Others	296 (5.8)	3 (11.1)	
Spondyloarthropathies (*n* (%))			
Ankylosing spondylitis	26 (0.5)	3 (11.1)	< 0.001*
Enteropathic arthritis (UC)	46 (0.9)	3 (11.7)	0.002**
Enteropathic arthritis (CD)	15 (0.3)	2 (7.1)	0.002**
Smoking status (*n* (%))			
Current smoker	1266 (24.8)	6 (22.2)	0.1
Former smoker	1026 (20.1)	10 (37.0)	
Never smoker	2814 (55.1)	11 (40.7)	
Current health status (*n* (%))			
Mental health not good past 30 days^b^	2798 (54.8)	13 (48.2)	0.5
Felt anxious past 30 days^c^	3533 (69.2)	21 (77.8)	0.3
Educational level (*n* (%))			
Less than 9th grade	463 (10.3)	1 (4.5)	0.2
9th grade to less than high school graduate	687 (15.3)	4 (18.2)	
High school graduate or GED equivalent	1044 (23.2)	4 (18.2)	
Some college	1335 (29.6)	4 (18.2)	
College graduate and beyond	973 (21.6)	9 (40.9)	
Annual house income (*n* (%))			
< US$20,000/year	958 (21.3)	2 (9.1)	0.4
US$20,000–44,999/year	1422 (31.6)	10 (45.5)	
US$45,000–74,999/year	893 (19.8)	5 (22.7)	
≥ US$75,000/year	1229 (27.3)	5 (22.7)	

^a^All *p* values are unadjusted. *p* values were calculated using the Wald test for continuous variables and the design-adjusted Rao–Scott–Pearson *χ*^2^ test for categorical variables

^b^For how many days during the past 30 days was your mental health not good (which includes stress, depression, and problems with emotions)?

^c^During the past 30 days, for about how many days have you felt worried, tense, or anxious? (Any number of days reported in these two questions was considered to be a positive finding)

*Significant at the 0.001 probability level

**Significant at the 0.01 probability level

**Table 2:** Multivariate analysis of self-reported uveitis in the arthritis population

Variable	OR 95% CI	*p* value
Age	1.04 (1.01–1.07)	0.02
Female sex	2.07 (0.79–5.44)	0.13
Ethnicity^a^	1.92 (0.69–5.33)	0.20
Ever smoker	3.18 (1.59–6.37)	0.003**
Never smoker	1.00	
Former smoker	4.26 (2.19–8.26)	< 0.001*
Current smoker	1.97 (0.59–6.52)	0.25
Ankylosing spondylitis	16.64 (3.64–76.09)	0.001*
Ulcerative colitis	11.34 (2.69–47.88)	0.003**
Crohn’s disease	22.16 (2.64–186.17)	0.007**
Mental health	1.02 (0.96–1.07)	0.55
Anxiety	1.04 (0.99–1.09)	0.12

Multivariate variable is corrected for age and gender

^a^Reference group for ethnicity: non-Hispanic whites

*Significant at the 0.001 probability level

**Significant at the 0.01 probability level

Of the 27 participants with uveitis, 63% were female and 37% were male, compared with 51.5% of females in the non-uveitis group. The OR for female sex using multivariate analysis was of 2.07 (95% CI 0.79–5.44; *p =* 0.1). The mean age for the uveitis population was of 53 ± 13 years and 44 ± 14 years for the non-uveitis one. This difference showed an OR in years of age of 1.04 (95% CI 1.01–1.07; *p* = 0.02) by multivariate analysis, indicating higher odds of uveitis with greater age.

The ethnic distribution included 8 Hispanics (29.6%), 10 Caucasians (37%), 6 African Americans (22.2%), and 3 Others (11.1%) in the uveitis group. No significant association was found for any specific subgroup neither in the univariate nor in the multivariate analysis when compared with the non-uveitis group.

With regard to the presence of different spondyloarthropathies, 3 patients (11.1%) from the uveitis group self-reported a diagnosis of AS, in comparison with 26 (0.5%) from the non-uveitis group. In both the univariate and multivariate analyses, we found that the presence of a diagnosis of AS in the uveitis group was statistically significant (*p* ≤ 0.001), with an OR of 16.64 (95% CI 3.64–76.09) using multivariate analysis. Equally significant was the presence of UC and CD among the participants with uveitis, with 3 (11.7%) and 2 (7.1%) reporting each diagnosis, respectively. The OR of the multivariate analysis was 11.34 (95% CI 2.69–47.88; *p* = 0.003) for UC and 22.16 (95% CI 2.64–186.17; *p* = 0.007) for CD.

Sixteen (59.2%) out of the 27 uveitis participants reported having smoked at least 100 cigarettes in their lifetime, compared with 2292 (44.9%) from the non-uveitis group. The multivariate analysis for a positive smoking history in the uveitis group, adjusting for age and sex, showed an OR of 3.18 (95% CI 1.59–6.37; *p* = 0.003), using non-smokers as the comparator. Of the 16 participants with a positive smoking history from the uveitis group, 6 (37.5%) were current smokers and 10 (62.5%) former smokers, leaving just 11 (40.7%) never smokers. In the non-uveitis group, those numbers were 1266 (24.8%) for the current smokers, 1026 (20.1%) for the former smokers, and 2814 (55.1%) for the never smokers. Multivariate analysis of smoking status in the uveitis group demonstrated that former smokers had higher odds of uveitis than current smokers when compared with non-smokers, with an OR of 4.26 (95% CI 2.19–8.26; *p* <  0.001) and 1.97 (95% CI 0.59–6.52; *p* = 0.25), respectively.

Subanalyses for mental health and stress values did not show any statistically significant result, with 48.2% of the participants with diagnosed uveitis reporting poor mental health for at least 1 day during the past 30 days (*p* = 0.5) and 77.8% reporting anxiety or worriedness for at least 1 day during the past 30 days (*p* = 0.3). Likewise, educational level and annual household income were not found to have any association with a diagnosis of uveitis, with *p* values of 0.2 and 0.4, respectively.

Vitamin D levels (25-dehydroxyvitamin D2 + D3) were measured in serum samples, and 25 out of the 27 patients with uveitis had the laboratory test performed. None of the 25 patients were found to have values indicative of deficiency (less than or equal to 30 nmol/L). The mean levels for vitamin D in the uveitis group were 65.3 ± 25 nmol/L and 61.6 ± 25 nmol/L in the non-uveitis group (*p* = 0.7).

## Discussion

In our US population-based study using the NHANES database, we found that AS, cigarette smoking, and higher age were positively associated to self-reported uveitis. Its main strengths include the wide diversity of the database in terms of geographic characteristics and socioeconomic status and its representation of the national noninstitutionalized population, making it the first nationwide study in the country comparing the presence of self-reported uveitis with different demographic and clinical factors.

For our analysis, we looked at known risk factors for the development of uveitis, such as, UC, and CD. It has been previously reported that HLA-B27-positive spondyloarthritides have the highest association with non-infectious uveitis in the adult population in North America and Europe [5, 13–15]. In our study, we found a statistically significant predominance of all three diagnoses in the uveitis group compared with the non-uveitis group, which supports the results from the previous literature.

Given that all analyses on our population-based study accounted for the multi-stage nature of NHANES, these results are nationally representative, and thus, we can extrapolate them in order to obtain an estimate of the actual prevalence of the disease in the country, which is of 5.4 per 1000 residents (95% CI 3.4–8.5/1000). This number differs from previous epidemiologic studies carried out by Thorne et al. [12] and Gritz and Wong [18], who reported an overall prevalence of 121 cases per 100,000 adults (95% CI 117.5–124.3) in the USA and 115.3 per 100,000 persons in Northern California, respectively. If we would only consider the patients from our database who reported having received treatment for uveitis (*n* = 19) as a way of confirming their diagnoses, the prevalence estimate in our population would still be higher than those previously mentioned (3.4/1000 persons; 95% CI 2.3–5.1/1000). A possible explanation could be that our study is not limited to a specific type of uveitis, as it is the one from Thorne et al., which mainly looked at the prevalence of noninfectious uveitis, and that it is based on the national population, as opposed to Gritz and Wong’s study, which may give more regional results based on Northern California population, and Thorne et al., who only studied privately insured patients, excluding an important part of society. Also, those two studies calculated the prevalence of the disease during a 1-year period, while we measured the lifetime prevalence of the participants, so age may be playing a role in this prevalence estimate as a risk factor for the disease.

Prior studies have shown that ocular inflammatory disorders have a higher incidence in women compared with men, particularly in women of childbearing age, and that this gender difference increases with increasing age [17]. Sex hormones and the presence of an extra X chromosome in women are thought to play important roles in the development of these immune-mediated diseases [17, 35, 36]. While this gender difference has been found in studies performed in several developed countries [5, 6], a reverse gender predilection has been noted in developing countries, such as India or Turkey, where a male predominance was found [14, 15, 19–21]. In our study which examined residents in the USA, a slight female predominance was found, although it was not statistically significant.

As previously mentioned, a positive association between age and the development of uveitis was found in our analysis, where the greater the age, the higher the odds of suffering from uveitis. This finding supports results obtained in the Pacific Ocular Inflammation Study carried out in a Hawaiian population and Gritz and Wong’s Northern California Epidemiology of Uveitis Study [1, 18]. Several other studies have reported the highest incidence of uveitis in persons between the ages of 20 and 40 years [10, 14, 19–23]. This differs from our results, which showed a higher mean age of 53 ± 13 years old, with most of the patients concentrated between the ages of 40 to 60 years. However, our population participated in a survey that asked “Have you ever been diagnosed with uveitis?”, which may be the reason for this discordance, since the older the participants, the higher the chance for them to have had uveitis in the past. The age of diagnosis was not an available variable on the dataset. Although age has been a risk factor that has not been unanimously related with uveitis, our study adds evidence of age being a related risk factor in a large-scale population-based study.

Our analysis of ethnicity did not reveal any specific ethnic group predominance, which may be partly due to the small sample size or a true lack of correlation. Our results contrast with other studies that have shown slight group differences, including the Pacific Ocular Inflammation Study which found a higher incidence of uveitis in the white population when compared with Asians and Pacific Islanders [1]. Furthermore, Nguyen et al. reported a higher incidence of uveitis in African American patients with inflammatory bowel disease in comparison with Caucasian patients [37].

A link between vitamin D deficiency status and uveitis has also been previously reported. A study done in China in 2010 looking at patients with Vogt–Koyanagi–Harada (VKH) disease described a possible association between VKH and vitamin D deficiency [28]. A more recent study carried out in the Massachusetts Eye and Ear Infirmary determined that there is a correlation between vitamin D deficiency and non-infectious uveitis [27]. All patients in our study population had normal levels of vitamin D in serum samples, which differs from the results of these two previous studies.

Our present study also demonstrates that a positive history of cigarette smoking is associated with higher odds of a uveitis diagnosis. This higher rate of uveitis was obtained through the former smokers’ group, leading us to theorize that patients stopped smoking when they were diagnosed with the disease due to their doctor’s recommendation. This association between uveitis and tobacco smoking supports the results obtained by Lin et al., who concluded that cigarette smoking is a risk factor for all anatomic types of uveitis as well as infectious uveitis, and Yuen et al., who found that cigarette smoking had a strong association mainly with noninfectious uveitis [24, 25]. It has also been found that tobacco smokers have a higher risk for active disease, the need for higher doses of steroids in order to control their uveitis, an earlier development of the disease, higher incidence of bilateral ocular inflammation, higher odds of disease recurrence, and higher rates of cystoid macular edema as a complication [26, 38, 39]. This positive association between tobacco smoking and uveitis, however, may not apply to all forms of uveitis because tobacco smoking does not have any negative ocular effects in patients with Behçet’s disease [40].

Cigarette smoking is the leading preventable cause of death in the USA [41]. It causes more than 480,000 deaths each year in the USA and accounts for nearly one in five deaths [41–43]. Chronic exposure to tobacco smoke or nicotine has an effect on the immune system, increasing the release of pro-inflammatory cytokines such as TNF-α, IL-1, IL-6, IL-8, and GM-CSF, as well as decreasing the release of anti-inflammatory ones, such as IL-10. These findings support the theories that cigarette smoking has a positive correlation with the development of many chronic inflammatory and autoimmune diseases, although the causal and pathophysiological relationship still remains unclear [44–51].

Despite the advantages of using such a large database, our study has several limitations intrinsic of the NHANES design. Some of these weaknesses are the small sample size of those with uveitis, which may have underpowered the study and thus prevent us from identifying well-known disease predictors, such as gender; the fact that it is generalizable only to the noninstitutionalized US residents, excluding the institutionalized population in the country; and the cross-sectional nature of the study, which precludes the analysis of the pathology over time. This can also lead to an overestimation of the prevalence and risk factors.

Moreover, self-report of uveitis by the participants may refer to either an acute isolated attack or to chronic disease, which is not specified in the questionnaires. The questionnaire also does not ask the participants to specify the location of the uveitis (anterior, intermediate, posterior, or panuveitis). It is also possible that, since the diagnosis of uveitis was not verified by an ophthalmologist, patients may have confused their diagnosis with other types of eye inflammation. The fact that only 19 out of 27 patients with uveitis reported having received treatment raises suspicion on the accuracy of the diagnosis, since it is not the standard of care to leave uveitis untreated. On the other hand, uveitis may not have been diagnosed in all participants. Some participants may not have been aware of a uveitis episode and therefore not reported it. Therefore, recall bias may influence the results and add more uncertainty to the prevalence estimate. A study published in 1991 suggested that self-reported ocular disease should not be the only source of information for prevalence estimates and that clinical determinations are necessary [52], and a more recent paper trying to determine the validity of self-reported eye disease in a Latino population concluded that the sensitivity is generally very low, so there is a high chance that patients who did not report the disease actually had it, while the specificity was high for all the diagnoses [53]. However, none of these two studies asked for a diagnosis of uveitis, and only self-reported cataract, glaucoma, diabetic retinopathy or macular degeneration were studied. There is no evidence in the literature of the validity of self-reported uveitis for epidemiologic studies.

Other limitations of the study may be that when looking at the smoking history, we just focused on the general categories of whether patients were never, former, or current smokers; we did not evaluate specific quantities such as number of cigarettes smoked per day, years of smoking, or the timing of smoking in relation to the diagnosis of uveitis; and the fact that correlation between two variables does not prove causation [54, 55]. Moreover, the 95% confidence intervals of multivariable models were wide, indicating that there may be some imprecision in the effect estimates.

## Conclusions

In summary, our analysis of a sample of the US adult population extracted from the NHANES database identified a positive association between self-reported uveitis and the presence of AS, UC, and CD, a positive smoking history, and higher age. Further prospective and longitudinal studies and investigations with different demographic populations would aid in our understanding of the roles of these risk factors in the development of uveitis.

## Methods

### NHANES database analysis

For our study, we used a sample of the US population extracted from the database of the NHANES for the years 2009 and 2010. The NHANES is a source of cross-sectional information about the health and nutritional status of the American population obtained through questionnaires about demographics, socioeconomic status, diet, and health, as well as physical examination. The survey examines approximately 5000 persons each year in counties across the USA. It is a major program of the National Center for Health Statistics (NCHS) and is administered by the Centers for Disease Control and Prevention (CDC) in order to provide US health statistics.

It uses a complex, multistage probability sampling design in order to select a sample representative of the civilian noninstitutionalized household population of the country. A weighting scheme is applied in order to more accurately estimate disease prevalence in the USA [56]. Since this research uses publicly available de-identified data, it is considered exempt from the Committee for Human Research approval by the University of California, San Francisco.

We analyzed the data to assess the association between self-reported uveitis and various demographic and clinical data. The sample consisted of 5106 American residents between the ages 20 and 69 years old who were asked for a diagnosis of uveitis through the question “Has a doctor or other health professional ever told you that you had iritis or uveitis?” Predictor values for that population group were the presence of a diagnosis of AS, UC, or CD, assessed by the questions, “Has a doctor or other health professional ever told you that you had ankylosing spondylitis/ulcerative colitis/Crohn’s disease?”; ethnicity (Mexican, other Hispanic, non-Hispanic white, non-Hispanic black, and Others); smoking history as determined by the answer to the question, “Have you smoked at least 100 cigarettes in your entire life?”; current smoking status based on the question, “Do you now smoke cigarettes?”; self-reported stress values collected through two questions asking the participants, “Now thinking about your mental health, which includes stress, depression, and problems with emotions, for how many days during the past 30 days was your mental health not good?” and “During the past 30 days, for about how many days have you felt worried, tense, or anxious?”; vitamin D serum levels; annual household income; and educational level.

For the multivariate analysis, the confounders taken into account were the ones with statistical significance in the univariate analysis and a strong association with the epidemiology of the disease—age and gender. The main outcome was considered to be a clinical diagnosis of uveitis based on the participant’s answer to the question above.

### Statistical analysis

We used RStudio (version 0.99.903, RStudio, Inc., Boston, MA) for our statistical analyses. NHANES is a complex designed survey which takes into account non-response rate and post-stratification. When a sample is weighted in NHANES, it is representative of the US census noninstitutionalized civilian population. In order to produce adequate statistical reliability, analysis was performed using weighting for subsets of the data. The distribution of possible confounding variables was compared between the participants with and without uveitis diagnosis using Rao–Scott–Pearson *χ*^2^ and Wald tests for categorical and continuous variables, respectively.

A multivariate logistic regression model analysis was performed in order to determine the odds of self-reported uveitis among the different ethnic groups, the presence or absence of a diagnosis of ankylosing spondylitis, a positive smoking history, and the mental health and stress values, adjusting for the confounding variables: age and gender. Additional sub-population analyses were done regarding the association between uveitis and vitamin D deficiency, annual household income, and educational level. The calculation of the prevalence estimate of uveitis in our population was survey-weighted. All analyses are accounted for the complex multi-stage design of the NHANES.

## Abbreviations

AS: Ankylosing spondylitis
NHANES: National Health and Nutrition Examination Survey
